# Supplementary material for: Prevalence and determinants of under-nutrition among children on ART in Ethiopia: A systematic review and meta-analysis
Source: PLoS One. 2024 Jun 20;19(6):e0303292. doi: 10.1371/journal.pone.0303292 (PMC11189179; doi:10.1371/journal.pone.0303292)
Supplement: S1 Table — (DOCX) [file pone.0303292.s002.docx]

S 2 Table: Risk of bias assessment for the included studies using the Hoy 2012 tool

| Studies | External validity | | | | Internal validity | | | | | |  | |
| --- | --- | --- | --- | --- | --- | --- | --- | --- | --- | --- | --- | --- |
|  | Representativeness s of the target population | Representativeness s of the sampling frame | Radom sampling g or census | Minimal responses bias | Data were collected directly | Acceptable case definition used in the study | Valid and reliable measurement | The same mode of data collection for all study subject | Appropriate length of prevalence period for parameter of interest | Appropriate numerators and denominator s of interest | No of yes | **Summary of risk of bias**  **assessment** |
| Haileselassie etal | Yes | Yes | yes | No | Yes | No | Yes | Yes | Yes | Yes | 8 | Low-risk |
| Tiruneh etal | Yes | Yes | yes | No | Yes | No | Yes | Yes | Yes | Yes | 8 | Low- risk |
| Gezahegn etal | Yes | Yes | yes | No | Yes | Yes | Yes | Yes | Yes | Yes | 9 | Low – risk |
| Jeylan etal | Yes | Yes | No | Yes | Yes | Yes | Yes | Yes | Yes | Yes | 9 | Low- risk |
| Megabiaw etal | Yes | Yes | Yes | Yes | Yes | No | Yes | Yes | Yes | Yes | 9 | Low- risk |
| Kedir etal | Yes | Yes | yes | No | Yes | Yes | Yes | Yes | Yes | Yes | 9 | Low- risk |
| Abdulkadir | Yes | Yes | Yes | Yes | No | No | Yes | Yes | Yes | Yes | 8 | Low- risk |
| Tiruneh etal | Yes | Yes | No | Yes | Yes | Yes | Yes | No | Yes | Yes | 8 | Low- risk |

| Teklemariam etal | Yes | Yes | Yes | Yes | No | Yes | Yes | No | Yes | Yes | 8 | Low- risk |
| --- | --- | --- | --- | --- | --- | --- | --- | --- | --- | --- | --- | --- |
| Mengist etal | Yes | Yes | Yes | Yes | No | No | Yes | Yes | Yes | Yes | 8 | Low-risk |
| Kusum Lata | Yes | Yes | No | Yes | Yes | Yes | Yes | Yes | Yes | Yes | 9 | Low- risk |
| Shiferaw and Gebremedhin | Yes | Yes | Yes | No | Yes | No | Yes | Yes | Yes | Yes | 8 | Low-risk |
| Sewale etal | Yes | Yes | Yes | Yes | Yes | Yes | Yes | No | Yes | Yes | 9 | Low-risk |
| Tekleab etal | Yes | Yes | Yes | Yes | Yes | Yes | No | Yes | Yes | Yes | 9 | Low- risk |
| Kebede etal | Yes | Yes | No | Yes | Yes | Yes | Yes | Yes | Yes | Yes | 9 | Low risk |
| Dessalegn et al | Yes | Yes | Yes | Yes | Yes | Yes | Yes | Yes | No | No | 8 | Low- risk |

Risk of bias assessment tool: Yes (low risk); No (high risk)

1. Representation: Was the study population a close representation of the national population?

2. Sampling: Was the sampling frame a true or close representation of the target population?

3. Random selection: Was some form of random selection used to select the sample OR was a census undertaken?

4. Non-response bias: Was the likelihood of non-response bias minimal?

5. Data collection: Were data collected directly from the subjects?

6. Case definition: Was an acceptable case definition used in the study?

7. Reliability and validity of study tool: Was the study instrument that measured the parameter of interest show to have reliability and validity?

8. Data collection: Was the same mode of data collection used for all subjects?

9. Prevalence period: Was the length of the prevalence period for the parameter of interest appropriate?

10. Numerators and denominators: Were the numerator(s) and denominator(s) for the parameter of interest appropriate?
