# Supplementary material for: Prevalence and determinants of under-nutrition among children on ART in Ethiopia: A systematic review and meta-analysis
Source: PLoS One. 2024 Jun 20;19(6):e0303292. doi: 10.1371/journal.pone.0303292 (PMC11189179; doi:10.1371/journal.pone.0303292)
Supplement: S2 Table — (DOCX) [file pone.0303292.s003.docx]

| **S 3 Table:** Shows the quality score of each study using Newcastle-Ottawa Scale (NOS) quality assessment tool adapted for cross-sectional and cohort studies. | | | | | | | | | | |
| --- | --- | --- | --- | --- | --- | --- | --- | --- | --- | --- |
| Authors | | Selection(Max*****) | | | | Comparability (Max**) | Outcomes(Max***) | | | Total  score |
|  | | Representiveness of the Sample | Sample Size | Non-  respondents | Ascertainment of the exposure (risk factor |  | Assessment | | Statistical  test |  |
| Cross-sectional studies | | | | | | | | | |  |
| Haileselassie et al. | | * | * | * | – | * | ** | | * | 7/10 |
| Tiruneh et al. | | * | * | - | ** | - | ** | | * | 7/10 |
| Gezahegn et al. | | * | * | – | ** | ** | ** | | * | 9/10 |
| Jeylan et al. | | * | * | - | ** | * | ** | | * | 8/10 |
| Megabiaw et al. | | * | * | - | ** | ** | ** | | * | 9/10 |
| Abdulkadir | | * | - | * | * | * | ** | | * | 710 |
| Teklemariam et al. | | * | - | - | ** | ** | * | | * | 7/10 |
| Tiruneh et al. | | * | * | * | * | ** | * | | * | 8/10 |
| Mengist et al. | | * | * | – | ** | * | ** | | * | 8/10 |
| Lata et al. | | * | * | – | ** | ** | * | | * | 8/10 |
| Shiferaw et al. | | * | * | * | * | * | * | | * | 7/10 |
| Sewale et al. | | * | * | * | ** | ** | * | | * | 9/10 |
| Dessalegn etal. | | * | * | - | ** | ** | * | | * | 8/10 |
| Cohort studies | | | | | | | | | | |
|  | Selection | | | | | Comparability | Outcomes | | | |
|  | Representiveness of exposed cohort | | Selection of non-exposed cohort | Ascertainment of exposure | Outcome not present at the start of the study |  | Assessment | Length of follow-up | Adequacy of follow-up |  |
| Kedir etal | * | | * | – | * | * | * | * | * | 7/9 |
| Tekleab etal | * | | * | * | * | ** | * | * | * | 9/9 |
| Kebede etal | * | | * | – | * | ** | * | * | * | 8/9 |
