# Supplementary material for: Prevalence and determinants of under-nutrition among children on ART in Ethiopia: A systematic review and meta-analysis
Source: PLoS One. 2024 Jun 20;19(6):e0303292. doi: 10.1371/journal.pone.0303292 (PMC11189179; doi:10.1371/journal.pone.0303292)
Supplement: S3 Table — (DOCX) [file pone.0303292.s004.docx]

**S4 Table:** Sensitivity analysis for the pooled prevalence of stunting in Ethiopia, from 2012-2022.

------------------------------------------------------------------------------

Study omitted Estimate [95% Conf. Interval]

-------------------+----------------------------------------------------------

Haileselassie etal 35.090965 23.111734 47.070194

Tiruneh etal 36.698181 25.723251 47.673107

Gezahegn etal 34.570568 22.677143 46.463993

Jeylan etal 36.045586 23.976931 48.114243

Megabiaw etal 31.706417 21.132271 42.280563

Abdulkadir 33.290558 21.813622 44.76749

Mengist etal 36.265011 24.405056 48.124966

Kusum Lata 32.087029 21.581324 42.592739

Shiferaw 34.382252 22.664295 46.100208

Sewale etal 33.370377 21.889193 44.851563

Tekleab etal 31.206978 20.680002 41.733952

Kebede etal 35.385311 22.928169 47.842457

Dessalegn et al 35.535751 23.535717 47.535789

-------------------+----------------------------------------------------------

Combined 34.279612 23.22652 45.332703

------------------------------------------------------------------------------
