# Supplementary material for: Prevalence and determinants of under-nutrition among children on ART in Ethiopia: A systematic review and meta-analysis
Source: PLoS One. 2024 Jun 20;19(6):e0303292. doi: 10.1371/journal.pone.0303292 (PMC11189179; doi:10.1371/journal.pone.0303292)
Supplement: S4 Table — (DOCX) [file pone.0303292.s005.docx]

**S5 Table:** Sensitivity analysis for the pooled prevalence of wasting in Ethiopia, from 2012-2022.

------------------------------------------------------------------------------

Study omitted Estimate [95% Conf. Interval]

-------------------+----------------------------------------------------------

Haileselassie etal 20.25326 12.875665 27.630854

Tiruneh etal 19.17939 12.838517 25.520264

Megabiaw etal 23.120121 17.110739 29.129501

Abdulkadir 19.932085 12.66355 27.200619

Teklemariam etal 22.547071 14.996519 30.097624

Mengist etal 20.424547 12.951478 27.897615

Shiferaw and

Gebremedhin 21.305145 13.520549 29.089741

Tekleab etal 21.764627 13.999204 29.53005

Kebede etal 21.788389 13.162413 30.414364

-------------------+----------------------------------------------------------

Combined 21.146187 14.174496 28.117878
