# Supplementary material for: Prevalence and determinants of under-nutrition among children on ART in Ethiopia: A systematic review and meta-analysis
Source: PLoS One. 2024 Jun 20;19(6):e0303292. doi: 10.1371/journal.pone.0303292 (PMC11189179; doi:10.1371/journal.pone.0303292)
Supplement: S5 Table — (DOCX) [file pone.0303292.s006.docx]

**S6 Table:** Sensitivity analysis for the pooled prevalence of Under-weight in Ethiopia, from 2012-2022.

------------------------------------------------------------------------------

Study omitted Estimate [95% Conf. Interval]

-------------------+----------------------------------------------------------

Jeylan etal 28.14856 20.596384 35.700737

Megabiaw etal 25.876442 19.31609 32.436794

Abdulkadir 26.116787 19.265564 32.96801

Tiruneh etal 27.438707 19.97835 34.899063

Teklemariam etal 29.491508 22.861177 36.121838

Mengist etal 28.789122 21.629574 35.948669

Kusum Lata 25.857578 19.569246 32.145908

Tekleab etal 26.187101 19.335911 33.038292

Kebede etal 28.378967 20.650303 36.107632

Dessalegn et al 28.435896 21.018137 35.853657

-------------------+----------------------------------------------------------

Combined 27.471553 20.803651 34.139456

------------------------------------------------------------------------------
