# Supplementary material for: Prevalence and determinants of under-nutrition among children on ART in Ethiopia: A systematic review and meta-analysis
Source: PLoS One. 2024 Jun 20;19(6):e0303292. doi: 10.1371/journal.pone.0303292 (PMC11189179; doi:10.1371/journal.pone.0303292)
Supplement: Attachment — Submitted filename: Response to Reviewers -2nd.docx [file pone.0303292.s008.docx]

**Dear Editor of PLOS ONE editorial office**

This is a point-by-point response letter which supplements the responses of authors to reviewers’ comments regarding to our manuscript. I am delighted to resubmit the revised version of our manuscript entitled “**Prevalence and determinants of under-nutrition among children on ART in Ethiopia: a systematic review and meta-analysis”** by Belete Gelaw Walle*1, Nigusie Selomon2, Chalie Marew Tiruneh2, Bogale Chekole5, Amare Kassaw2, Moges Wubneh3, Yibeltal Assefa5, and Kelemu Abebe6. We have taken the academic editorial vital comments and views as an opportunity for further improvements of our manuscript as per the journal requirements.

It is well-known that the manuscript has been reviewed by peer reviewers and sent back to the authors for second minor revision and resubmission. We would like to take this occasion to recognize the reviewers for their valuable and constructive comments. The reviewers’ comments were insightful and assisted us to improve the quality of our manuscript significantly. We are so glad and gratified to work with you. The point-by-point responses for each of the reviewers’ comments are addressed in the following pages using a point by point format. The authors’ responses are also shown by the track changes in the revised version of our manuscript. **Our responses are written in blue font color.**

We look forward to hear the progress from you at your earliest convenience.

Kind regards

Belete Gelaw

(On the behalf of other authors)

**Comments to the Author**

The authors of this systematic review and meta-analysis have presented useful data to determine the pooled national burden of under-nutrition among children on ART in Ethiopia. Conversely, there is minor revision that the reviewer would like the authors to revise for further improvement of our manuscript.

Authors’ response: we are very glad to the reviewer’s appreciation of our efforts and commitment; and we have just provided our respective responses to each of the specific reviewer comments and concerns detailed below. Additionally, we have addressed the additional journal requirements with in our document accordingly.

**Journal Requirements:**

Please review your reference list to ensure that it is complete and correct. If you have cited papers that have been retracted, please include the rationale for doing so in the manuscript text, or remove these references and replace them with relevant current references. Any changes to the reference list should be mentioned in the rebuttal letter that accompanies your revised manuscript. If you need to cite a retracted article, indicate the article’s retracted status in the References list and also include a citation and full reference for the retraction notice.

[Note: HTML markup is below. Please do not edit.]

**Authors’ response**: We have revised the reference section to ensure that whither it is complete and correct or not. As a result, we found that references are correct, complete, and consistent with the strength of evidence and we didn’t made any new citation as well as deletion or remove from list of references.

**Reviewers' comments:**

**Reviewer's Responses to Questions**

**Comments to the Author**

1. If the authors have adequately addressed your comments raised in a previous round of review and you feel that this manuscript is now acceptable for publication, you may indicate that here to bypass the “Comments to the Author” section, enter your conflict of interest statement in the “Confidential to Editor” section, and submit your "Accept" recommendation.

Reviewer #1: All comments have been addressed

Reviewer #2: All comments have been addressed

2. Is the manuscript technically sound, and do the data support the conclusions?

The manuscript must describe a technically sound piece of scientific research with data that supports the conclusions. Experiments must have been conducted rigorously, with appropriate controls, replication, and sample sizes. The conclusions must be drawn appropriately based on the data presented.

Reviewer #1: Yes

Reviewer #2: Yes

3. Has the statistical analysis been performed appropriately and rigorously?

Reviewer #1: Yes

Reviewer #2: Yes

4. Have the authors made all data underlying the findings in their manuscript fully available?

The PLOS Data policy requires authors to make all data underlying the findings described in their manuscript fully available without restriction, with rare exception (please refer to the Data Availability Statement in the manuscript PDF file). The data should be provided as part of the manuscript or its supporting information, or deposited to a public repository. For example, in addition to summary statistics, the data points behind means, medians and variance measures should be available. If there are restrictions on publicly sharing data—e.g. participant privacy or use of data from a third party—those must be specified.

Reviewer #1: Yes

Reviewer #2: Yes

5. Is the manuscript presented in an intelligible fashion and written in standard English?

PLOS ONE does not copyedit accepted manuscripts, so the language in submitted articles must be clear, correct, and unambiguous. Any typographical or grammatical errors should be corrected at revision, so please note any specific errors here.

Reviewer #1: Yes

Reviewer #2: No

6. Review Comments to the Author

Please use the space provided to explain your answers to the questions above. You may also include additional comments for the author, including concerns about dual publication, research ethics, or publication ethics. (Please upload your review as an attachment if it exceeds 20,000 characters)

Reviewer #1: (No Response)

Reviewer #2: Dear authors

Thank you for your effort to address my questions and comments on the first draft of your manuscript. Here i kindly ask your effort to edit and revise your manuscript to fulfil the standard English.

**Authors’ response**: Thank you very much. We have carefully reviewed the manuscript and corrected any minor grammatical, typographic errors, and /inconsistencies. The changes can be appreciated from the tracked insertions and deletions on the second revised version of our manuscript.
